# Chronic Dermatographic Urticaria Secondary to Systemic Lupus Erythematosus

**DOI:** 10.7759/cureus.63109

**Published:** 2024-06-25

**Authors:** Miis Akel, Crystal Barroca, Alex Blanca, Shakil O Huq, Dhruv Ratra, Sahil Shah, Sergio Hernandez Borges

**Affiliations:** 1 Medicine, Dr. Kiran C. Patel College of Osteopathic Medicine, Nova Southeastern University, Clearwater, USA; 2 Cardiology, Dr. Kiran C. Patel College of Osteopathic Medicine, Nova Southeastern University, Clearwater, USA; 3 Osteopathic Medicine, Dr. Kiran C. Patel College of Osteopathic Medicine, Nova Southeastern University, Davie, USA; 4 Internal Medicine, Dr. Kiran C. Patel College of Osteopathic Medicine, Nova Southeastern University, Davie, USA; 5 Anesthesiology, Dr. Kiran C. Patel College of Osteopathic Medicine, Nova Southeastern University, Miami, USA; 6 Pain Management, Larkin Community Hospital Palm Springs Campus, Miami, USA; 7 Internal Medicine/Family Medicine, Larkin Community Hospital Palm Springs Campus, Miami, USA

**Keywords:** sle associated rash, dermatographia, hydroxyzine, hydroxychloroquine treatment, itchy rash, chronic dermatographic urticaria, systemic lupus erythromatosus

## Abstract

Systemic lupus erythematosus (SLE) poses significant challenges in diagnosis and management due to its diverse clinical manifestations, including various skin abnormalities. Chronic dermatographic urticaria, although less recognized, has been suggested to have an association with SLE; however, evidence supporting this connection remains limited. We present a case of chronic dermatographic urticaria secondary to SLE in a 26-year-old female. Despite ineffective conventional treatments, the initiation of hydroxyzine resulted in notable symptom improvement without adverse effects. This case underlines the importance of recognizing and addressing less common dermatological manifestations in SLE, emphasizing the need for a comprehensive approach to optimize patient outcomes. It highlights the potential utility of hydroxyzine in managing the refractory symptoms of chronic dermatographic urticaria in SLE patients. This report contributes to the expanding evidence regarding the complex interplay between SLE and dermatographic urticaria, necessitating further research to understand underlying mechanisms and establish optimal treatment strategies. Enhanced awareness and understanding of such associations are crucial for facilitating early diagnosis and tailored management approaches in patients with SLE, ultimately improving their quality of life and clinical outcomes.

## Introduction

Systemic lupus erythematosus (SLE) is a chronic autoimmune disorder characterized by its complex pathogenesis. Extensive effects impact different regions of the human body, including the skin, joints, kidneys, brain, and other organs. SLE induces widespread inflammation and autoantibody production, leading to a multifaceted clinical presentation. Dermatologically, SLE may manifest as malar rash, discoid lesions, or photosensitivity, each noted for its variability and diagnostic challenge [1]. At the cellular level, SLE involves abnormal immune response regulation with autoantibodies. Immune complexes are deposited into tissues, causing inflammation and damage. Histopathologically, SLE is characterized by interface dermatitis, deposition of mucin, and a lymphocytic infiltrate, which are key diagnostic features [2].

The prevalence of SLE is significantly higher in women, with nearly 10 women affected for every man [3], and it prominently occurs in younger individuals of Black, Asian, and Hispanic descent [3,4]. The clinical manifestation of SLE varies widely among individuals, with symptoms fluctuating in severity and presentation. Common symptoms include chest pain upon deep breathing, fatigue, unexplained fever, malaise, hair loss, weight fluctuation, mouth ulcers, photosensitivity, and the characteristic butterfly rash seen in approximately half of SLE patients [4,5]. Beyond cutaneous manifestations, SLE can involve multiple organs, including the brain, digestive tract, heart, lungs, kidneys, and blood vessels, necessitating a comprehensive and nuanced approach to management and treatment.

Chronic dermatographic urticaria is a unique form of urticaria characterized by persistent hives and itching triggered by minor physical stimuli such as pressure or scratching. The pathophysiology of this condition involves an exaggerated release of histamine and other vasoactive substances from mast cells in the skin upon stimulation. T cells also play a crucial role by modulating immune responses, which may overlap with the mechanisms seen in SLE [6,7]. Although not specific to lupus, urticaria, including chronic dermatographic urticaria, can occur in patients with both systemic and cutaneous lupus erythematosus, further complicating diagnosis and management. The histopathological findings in chronic dermatographic urticaria typically include dermal edema and a perivascular infiltrate of lymphocytes and eosinophils, distinct from but potentially overlapping with the pathology observed in SLE [8]. This underscores the need for detailed and careful differential diagnosis in clinical practice, highlighting the complex interplay between these autoimmune and allergic phenomena.

In this report, a case of chronic dermatographic urticaria secondary to SLE is presented to highlight the complex interplay between SLE and chronic dermatographic urticaria, emphasizing the necessity for meticulous evaluation and personalized therapeutic strategies in similar clinical contexts.

## Case presentation

**Figure 1:**
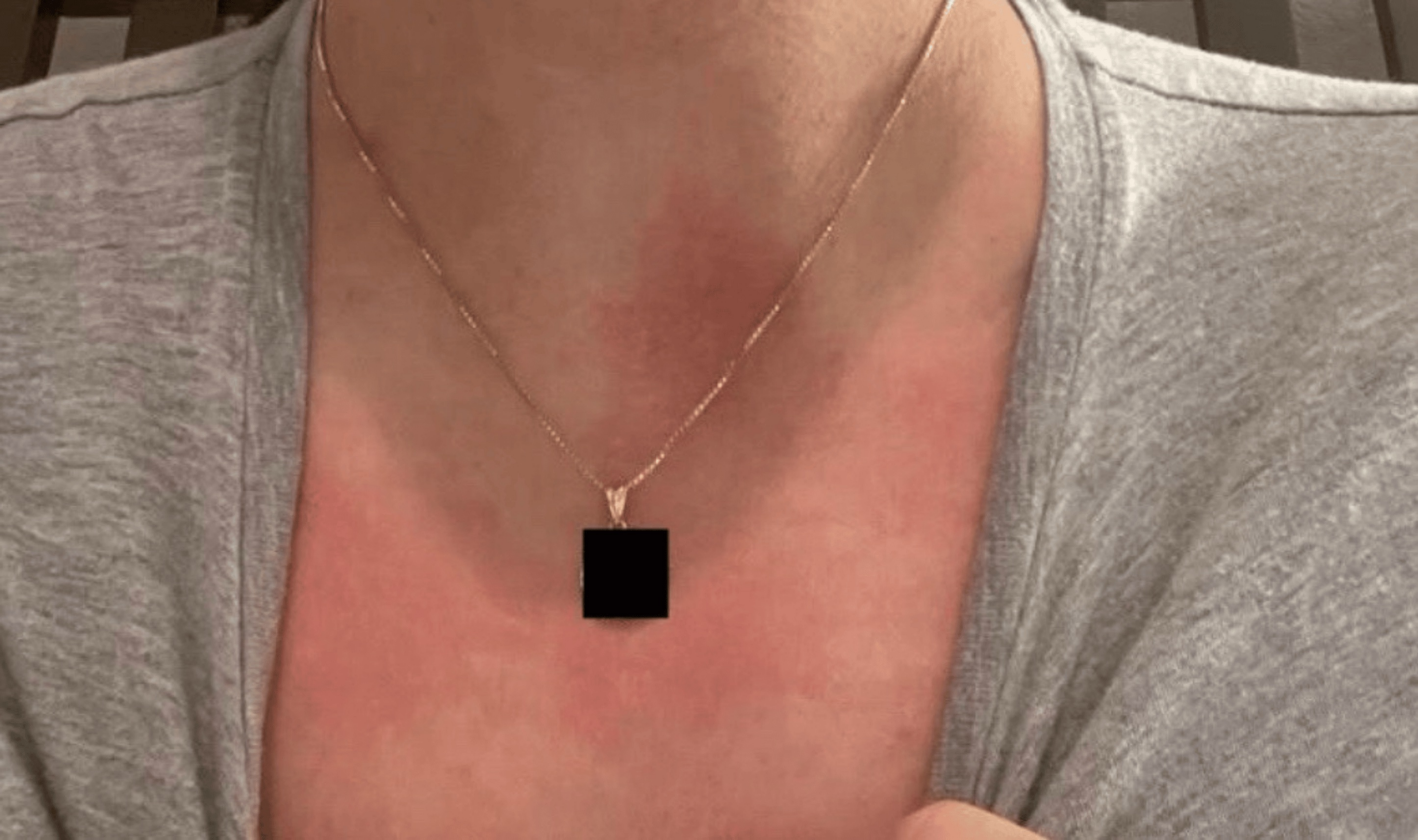
A pruritic, erythematous, macular rash with irregular borders on the anterior chest

**Figure 2:**
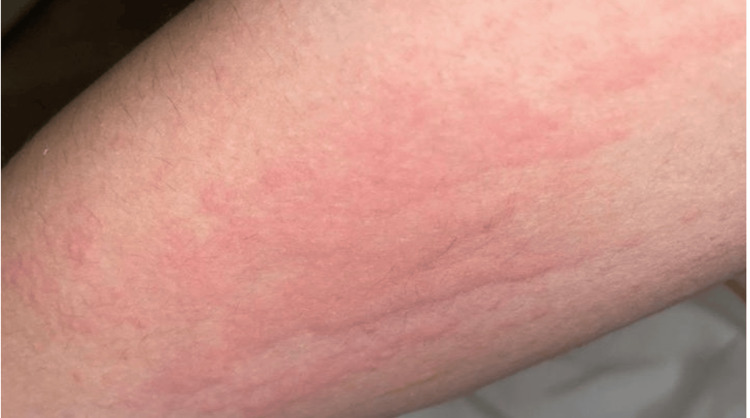
An erythematous, urticarial rash with irregular borders on the anterior arm

**Figure 3:**
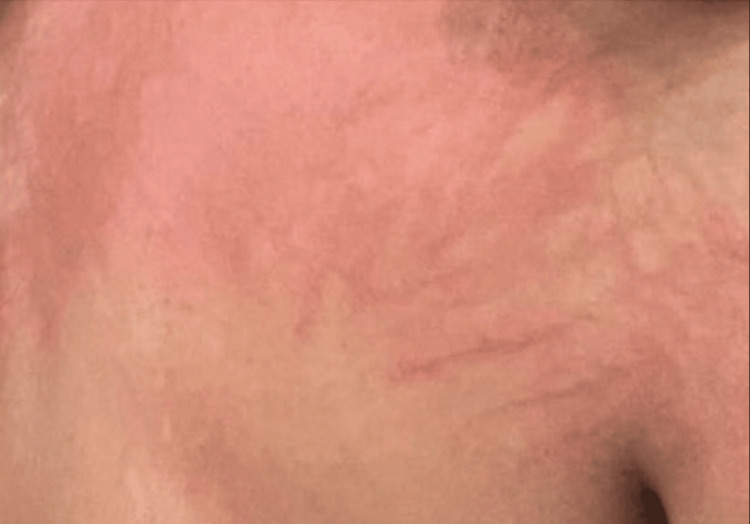
An urticarial rash with visible excoriations

A 26-year-old female with a past medical history of SLE presented with a two-week history of an itchy rash on her arms and trunk (Figures 1-3). Despite treatment with over-the-counter antihistamines, including cetirizine and diphenhydramine, the rash persisted, causing significant discomfort. The patient reported transient relief with cetirizine in the morning, followed by a recurrence of symptoms after a few hours, and used diphenhydramine at night for sleep, which provided only temporary relief. She was diagnosed with SLE four years ago, and her treatment course has included hydroxychloroquine 200 mg daily since her diagnosis. The patient has had multiple lupus flares within the past four years, occurring every three to four months, that were managed with methylprednisolone.

The patient recently moved apartments but continued to experience the rash even after staying at her parents’ house for a week. She denied changes in hygiene or personal care products (such as shampoos or laundry detergents). Her only changes in medication included antihistamines that she began in an effort to alleviate the rash. After two weeks of symptoms, she sought care at urgent care, where she received a 40 mg methylprednisolone injection, providing temporary relief for eight hours before symptoms recurred. Subsequently, she was referred to a dermatologist, who prescribed triamcinolone topical cream and a methylprednisolone pack. Despite these interventions, the rash persisted, and the patient developed insomnia and persistent itching. The patient stated that the pruritus and rash were concomitant and continuous, with antihistamines only providing temporary relief prior to the return of symptoms. She endorsed that she tries not to scratch her lesions, but the itching becomes unbearable. Given the patient’s medical history of SLE and the lack of response to conventional treatments, further evaluation was pursued to understand the underlying etiology.

Laboratory investigations, including allergy blood work, erythrocyte sedimentation rate (ESR), complete metabolic panel (CMP), and complete blood count (CBC), were conducted as shown in Table 1. Hepatitis A IgM, hepatitis B surface antigen, hepatitis B core antibody IgM, and hepatitis C antibody were nonreactive. CMP was non-pertinent. A triiodothyronine antibody was negative. A respiratory allergy panel was also negative, thus ruling out acute allergen hypersensitivities. Thyroid-stimulating hormone (TSH) with reflex to FT4 was within normal limits, with TSH measuring 1.4 mU/L within the normal range of 0.4-4.0 mU/L. The ESR was 6 mm/h, which is within normal limits (0-20 mm/h). CBC, including absolute monocytes, eosinophils, basophils, and lymphocytes, was within normal limits.

**Table 1:** Laboratory results presented include a comprehensive metabolic panel, CBC, and allergy profiles ALT: alanine aminotransferase; AST: aspartate aminotransferase; BUN: blood urea nitrogen; CBC: complete blood count; CMP: complete metabolic panel; D1: Dermatophagoides pteronyssinus; D2: Dermatophagoides farina; eGFR: estimated glomerular filtration rate; ESR: erythrocyte sedimentation rate; M1: Penicillium notatum; M2: Cladosporium herbarum; M3: Aspergillus fumigatus; T3: triiodothyronine; TSH: thyroid-stimulating hormone

Laboratory investigation	Result	Reference range
Allergy blood work	Negative	Negative
ESR	6 mm/h	0-20 mm/h
CMP		
Blood glucose	80 mg/dL	70-100 mg/dL
Creatinine	0.65 mg/dL	0.6-1.2 mg/dL
BUN	13 mg/dL	7-20 mg/dL
eGFR	104 mL/min/1.73m²	>60 mL/min/1.73m²
Potassium	4.2 mmol/L	3.5-5.1 mmol/L
Sodium	140 mmol/L	135-145 mmol/L
Chloride	104 mmol/L	98-107 mmol/L
Calcium	9.7 mg/dL	8.5-10.5 mg/dL
Carbon dioxide	27 mmol/L	23-29 mmol/L
Total protein	7.5 g/dL	6.0-8.5 g/dL
Albumin	4.7 g/dL	3.5-5.0 g/dL
Bilirubin	0.6 mg/dL	0.1-1.2 mg/dL
Alkaline phosphatase	40 U/L	40-150 U/L
AST	11 U/L	5-40 U/L
ALT	9 U/L	7-56 U/L
Hepatitis A IgM	Nonreactive	Nonreactive
Hepatitis B surface antigen	Nonreactive	Nonreactive
Hepatitis B core antibody IgM	Nonreactive	Nonreactive
Hepatitis C antibody	Nonreactive	Nonreactive
T3 antibody	Negative	Negative
Respiratory allergy panel	Negative	Negative
D1 IgE	<0.10 kU/L	<0.35 kU/L
D2 IgE	<0.10 kU/L	<0.35 kU/L
M1 IgE	<0.10 kU/L	<0.35 kU/L
M2 IgE	<0.10 kU/L	<0.35 kU/L
M3 IgE	<0.10 kU/L	<0.35 kU/L
Cat dander IgE	<0.10 kU/L	<0.35 kU/L
Dog dander IgE	<0.10 kU/L	<0.35 kU/L
Maple IgE	<0.10 kU/L	<0.35 kU/L
Birch IgE	<0.10 kU/L	<0.35 kU/L
Oak IgE	<0.10 kU/L	<0.35 kU/L
Food allergy profile		
Egg white IgE	<0.10 kU/L	<0.35 kU/L
Peanut IgE	<0.10 kU/L	<0.35 kU/L
Wheat IgE	<0.10 kU/L	<0.35 kU/L
Walnut IgE	<0.10 kU/L	<0.35 kU/L
Codfish IgE	<0.10 kU/L	<0.35 kU/L
Cow’s milk IgE	<0.10 kU/L	<0.35 kU/L
Soybean IgE	<0.10 kU/L	<0.35 kU/L
Shrimp IgE	<0.10 kU/L	<0.35 kU/L
Scallop IgE	<0.10 kU/L	<0.35 kU/L
Sesame seed IgE	<0.10 kU/L	<0.35 kU/L
Hazelnut IgE	<0.10 kU/L	<0.35 kU/L
TSH	1.40 mU/L	0.4-4.0 mU/L
CBC		
WBC	5.2 thousand/uL	4.0-11.0 thousand/uL
RBC	4.57 million/uL	4.20-5.80 million/uL
Hemoglobin	13.4 g/dL	12.0-16.0 g/dL
Hematocrit	39.80%	36.0-48.0%
Platelet count	293 thousand/uL	140-400 thousand/uL
Absolute neutrophils	2,543 cells/uL	1,500-7,800 cells/uL
Absolute lymphocytes	2,797 cells/uL	850-3,900 cells/uL
Absolute monocytes	472 cells/uL	200-950 cells/uL
Absolute eosinophils	59 cells/uL	15-500 cells/uL

Based on the clinical presentation, medical history of SLE, and laboratory findings, the patient was diagnosed with chronic dermatographic urticaria secondary to SLE. Other differentials considered include drug eruptions from hydroxychloroquine. The most common cutaneous side effects of hydroxychloroquine include rash, hyperpigmentation, and pruritus [5]; however, this condition usually presents as a gray-blue discoloration of skin and rashes that present similar to psoriasis, which was not seen in this patient.

The patient was initiated on hydroxyzine 100 mg to address both insomnia and skin reactions, in addition to continuing her medication regimen of hydroxychloroquine 200 mg qd and aspirin 81 mg qd for SLE management. The patient reported dramatic improvement in symptoms after two weeks of treatment with hydroxyzine. She denied pruritus or skin lesions and was advised to continue hydroxyzine for an additional 30 days, with a plan for follow-up to monitor her progress.

## Discussion

The absence of a definitive cure for SLE emphasizes the importance of managing the condition by controlling its symptoms. The potential severity of SLE’s manifestations, especially those affecting essential organs such as the heart, lungs, and kidneys, necessitates specialized treatment approaches [4,5,9]. Each individual with SLE requires a comprehensive evaluation to assess the disease’s activity, the specific organs involved, and the most appropriate treatment modality.

Therapeutic interventions for SLE encompass a spectrum of options tailored to address the unique needs of each patient. Nonsteroidal anti-inflammatory drugs, corticosteroids such as prednisone, and corticosteroid creams are commonly employed to alleviate symptoms and manage inflammation [10]. Additionally, disease-modifying antirheumatic drugs like hydroxychloroquine and methotrexate [11], as well as biologics such as belimumab and anifrolumab [12], may be prescribed to modulate the immune response and mitigate disease progression.

In the context of chronic dermatographic urticaria secondary to SLE, treatment strategies may diverge from conventional approaches. While over-the-counter antihistamines like cetirizine or diphenhydramine can provide symptomatic relief [13], hydroxyzine has demonstrated efficacy in numerous studies [14], particularly in reducing the need for long-term corticosteroid therapy, which carries potential adverse effects.

In the case presented, the patient was already receiving hydroxychloroquine to manage her SLE symptoms. The addition of hydroxyzine was motivated by its favorable effects on SLE-related manifestations such as rash, chronic inflammation, and insomnia. Although combining hydroxychloroquine and hydroxyzine carries a rare risk of serious side effects, such as the potential for irregular heart rhythms [15], a comprehensive risk-benefit assessment was undertaken to ensure the patient's safety.

Following the exhaustion of alternative treatment modalities, the incorporation of hydroxyzine proved to be beneficial in alleviating the patient’s symptoms without any discernible adverse effects. This highlights the importance of individualized treatment approaches in managing complex presentations of SLE, wherein the judicious selection of therapeutic agents can optimize outcomes while minimizing risks.

## Conclusions

This case emphasizes the importance of considering less common dermatological manifestations in patients with autoimmune diseases such as SLE. Chronic dermatographic urticaria, although rare, should be included in the differential diagnosis of persistent rashes in SLE patients, particularly when conventional treatments fail to provide relief. A comprehensive approach, including extensive clinical evaluation, appropriate laboratory investigations, and personalized management strategies, is essential for optimal patient care and outcomes.
